# One-year surveillance of *Chlamydia* spp. infection in stray cats from northeastern Italy

**DOI:** 10.3389/fvets.2025.1502642

**Published:** 2025-01-17

**Authors:** Laura Bellinati, Letizia Ceglie, Elisa Mazzotta, Mery Campalto, Laura Lucchese, Alda Natale

**Affiliations:** Istituto Zooprofilattico Sperimentale Delle Venezie, Legnaro, Italy

**Keywords:** *Chlamydiaceae*, *Chlamydia felis*, zoonosis, stray cats, conjunctivitis

## Abstract

Stray cats potentially act as reservoir for zoonotic agents, posing a risk of exposure to humans and domestic cats. The most prevalent *Chlamydiaceae* species in cats is *Chlamydia (C.) felis*, which is frequently associated with conjunctivitis and/or upper respiratory disease. The zoonotic potential of *C. felis* is believed to be relatively low, although exposure is possible through handling infected cats, by contact with their aerosol, and via fomites. Infection is more frequent in conditions of overcrowding, stress, poor hygiene and impairment of the immune system. For this reason, stray cats appear to be particularly susceptible to this pathogen. Aim of the study was to identify the molecular occurrence of *Chlamydiaceae* in stray and colony cats. Between May 2021 and June 2022, in seven provinces of northeastern Italy, veterinary services officers collected oropharyngeal swabs from 379 stray and colony cats. The samples were screened for *Chlamydiaceae* by real-time PCR targeting a 23S gene fragment. Positive samples were further analyzed either by a *C. felis*-specific qPCR or by amplification and sequencing of a 16S rRNA gene fragment. Overall, 7.7% of the cats tested positive for *Chlamydia* spp., and all were identified as *C. felis*. Among the positive individuals, only one exhibited respiratory symptoms. The analysis of anamnestic data revealed a significantly higher frequency of *C. felis* in male intact cats during the spring season, suggesting a potential behavioral aspect of this infection. Although the zoonotic risk of this *Chlamydia* species is low, it would be prudent to exercise caution when handling stray cats.

## 1 Introduction

The domestic cat (*Felis catus*) is one of the most common companion animals worldwide (1). Cats interact with humans in varying degrees, and could be classified as pets, stray/feral cats, or colony cats (2). Pet cats receive sustenance, shelter and medical care from their owner(s), whereas stray/feral cats move freely within urban/rural environments and may choose whether to socialize to people or not. If they are provided with food at one or more locations, they can become part of a ‘colony’, and eventually be colony cats (3).

Given that approximately three-quarters of emerging infectious diseases originate from animals (4), it is crucial to understand and monitor animal pathogens with zoonotic potential. The large populations of stray and colony cats commonly found in rural and urban areas need to be screened to identify the possible presence of such pathogens. Furthermore, these animals may come in contact with humans, free-roaming pets and other urban and peri-urban animals, emphasizing the need for a comprehensive surveillance program (5).

*Chlamydia* are obligate intracellular eubacterial pathogens that cause disease in humans and animals (6). The genus includes several species that are of significant public health concern, among which the human pathogens *C. trachomatis*, and *C. pneumoniae*, as well as zoonotic species such as *C. psittaci*, *C. abortus*, *C. caviae*, and *C. felis* (6).

*C. felis* infection is common in domestic cats and an important cause of feline acute and chronic conjunctivitis. The symptoms include eye discharge, conjunctival chemosis, blepharospasm, and redness of the nictitating membrane. Some cats may exhibit fever, lethargy, anorexia, sneezing, and nasal discharge (7). The infection often resolves spontaneously; however, in the event of an inadequate or absent treatment, or in case of a compromised immune system, the infection can result in chronic conjunctivitis. The duration of ocular shedding has been observed to last up to 60 days; however, intermittent shedding up to 8 months has also been documented in experimental cats, indicating the potential for an asymptomatic carrier state (7).

The zoonotic potential of *C. felis* is considered to be rather low, with rare reports of human conjunctivitis linked to infected cats (8–11). The human infection with *C. felis* typically does not result in serious illness, with only an HIV-positive patient whose infection was traced to a personal pet kitten (12) that presented chronic conjunctivitis and general malaise. Although the risk due to *C. felis* seems to be minimal, cat owners and professionals working with cats might be infected, and caution is advised when handling infected cats (11). Moreover, the presence of other zoonotic *Chlamydia* were reported in cats: *C. psittaci* was associated with a fatal outcome in a domestic cat (13), while *C. pneumoniae* was identified in cats with conjunctivitis (14).

The impact of *C. felis* and other respiratory pathogens in cats has been globally studied, revealing significant geographic and population-based variations. The prevalence of *C. felis* in domestic cats has been reported to range from 0 to 10% in clinically healthy animals and from 5.6 to 30.9% in cats with conjunctivitis (15–17). Several studies indicate that stray cats are more likely to be infected by *C. felis* than domestic cats (18–20). For instance, in 2010 a Slovakian study reported positivity rates of 30.9% in domestic cats and 65.78% in stray cats (19). This was confirmed in a more recent study in the same country, that reported high positivity rates in the population of stray cats (35.7%) and shelter cats (31%), whereas only 10% of domestic free-roaming cats resulted positive (17). In Switzerland *C. felis* positivity rates were 19.1% in stray cats and 11.6% in pets, with significant associations with conjunctivitis (18). In China, recent studies reported overall seroprevalence rates of 5.9% in cats and 12.1% in dogs, with seroprevalence rates of 3.9% in household cats and 14.3% in stray cats (21). Another Chinese study detected *C. felis* in 11.6% of stray cats (22). Research in Japan identified *C. felis* in 59.1% of cats with conjunctivitis (20), making it the most common pathogen associated with feline respiratory diseases in the country.

Overall, in stray cats the prevalence may reach 35.7%, while in subgroups of cats with conjunctivitis it can raise up to 65.8% (18, 23–28).

In Italy, data on the prevalence of *Chlamydiaceae* in stray cats remain scarce (29, 30) and suggest that *C. felis* is widely distributed among domestic and colony cats. The present study aimed to assess the prevalence of *Chlamydiaceae* in stray cats in northeastern Italy, and to identify the circulating chlamydial species. By filling this knowledge gap, our findings will contribute to a better understanding of *Chlamydia* epidemiology in unmanaged feline populations.

## 2 Materials and methods

### 2.1 Sample population

Between May 2021 and June 2022, oropharyngeal swabs were collected from 389 stray and colony cats in seven provinces of northeastern Italy (Bozen and Trento in the Trentino-Alto Adige region and Padua, Rovigo, Venice, Vicenza, and Verona in the Veneto region). Sampling was carried out under anesthesia by veterinarians in the context of “trap–neutered–return” (TNR) programs, in compliance with the Guide for the Care and Use of Laboratory Animals and Directive 2010/63/EU for animal experiments (national law: D.L. 26/2014). The study received the approval of the Ethical Committee of the Istituto Zooprofilattico Sperimentale delle Venezie—IZSVe (CE_IZSVE 8/2020). Dry swabs were maintained at +4°C and conveyed to the laboratory within 24 h. Upon arrival, they were stored at −80°C until further processing.

Oropharyngeal swabs were chosen over nasal or conjunctival swabs, typically more specific for *C. felis*, to optimize sampling for multiple diagnostic purposes as part of a broader research project funded by the Italian Ministry of Health (RC IZSVE 12/19). This approach enabled concurrent testing for pathogens such as *Capnocytophaga* spp., SARS-CoV-2, and Influenza A virus. Some results from this research have already been published (31, 32), while others are currently under submission.

The dataset included information on the sex, castration status, age, clinical symptoms, and geographical locations of the stray cats enrolled, along with the date and province of sample collection. Cats were categorized into four age groups (33): kittens (up to 1 year old), young adults (1 to 6 years old), mature adults (7 to 10 years old), and seniors (older than 10 years). To assess any correlation between infection and seasonality, samples were assigned to a specific seasons based on the collection date: spring (from March to May), summer (from June to August), autumn (from September to November), and winter (from December to February).

### 2.2 Molecular investigation

The collected oropharyngeal swabs were cut into sterile microtubes filled with 1 mL of 1X phosphate-buffered saline (PBS), mixed by vortexing, and stored at -80°C. DNA extraction was performed using the KingFisher^™^ Flex Purification System instrument (Life Technologies, Carlsbad, CA, United States) and the ID Gene^®^ Mag Universal Extraction Kit (IDvet, Grabels, France), according to the manufacturer’s instructions. A pretreatment step was performed before extraction, consisting of the addition of 20 μL of Proteinase K (QIAGEN, Hilden, Germany), 250 μL of the Lysis buffer provided by the kit, and 100 μL of the sample, incubated for 10 min at 70°C. Every DNA extraction included a negative control [nuclease-free water (Merk Life Science S.r.l., Darmstadt, Germany)]. DNA was stored at -80°C until further processing.

The eluted DNAs were screened with a *Chlamydiaceae* family-specific real-time PCR assay targeting a 111 bp fragment of the 23S rRNA gene (34). A universal heterologous control, Intype IC-DNA (Indical Bioscience GmbH, Leipzig, Germany), was added to each DNA with a ratio of 1:10 of the total elution volume and co-amplified (35), in order to validate each negative result (36). *C. felis* DNA and DNase-RNase free water were used as positive and negative controls for the reaction mix in each reaction run.

All *Chlamydiaceae-*positive samples (cut-off Ct ≤ 40) were further analyzed using a species-specific real-time PCR targeting a 78 bp fragment of the outer membrane protein of *C. felis* (37). Negative samples to the species-specific real-time PCR were further characterized by sequencing a 278 bp portion of the 16S rRNA gene (38).

Samples with a Ct > 35.0 in the *Chlamydiaceae* family-specific real-time PCR and negative in both species-specific assays were classified as negative. This decision was made taking into account possible cross-reactions with related microorganisms (36).

### 2.3 Statistical analysis

Data were analyzed using the open-source software R (version 4.3.3). The outcome variable was defined as the infection status of the animals. The prevalence of positive animals was calculated for each demographic and clinical data, along with the 95% confidence interval (95% CI). Statistical significance was assessed using Pearson’s χ^2^ test.

Generalized Additive Models (GAMs) were employed to evaluate the associations between predictor variables and the outcome variable. The analysis was conducted using the ‘mgcv’ package with a binomial error distribution (logit-link function). Odds ratios were calculated for the following predictors: sampling site, sampling season, sex (male *vs* female), castration status, age, and clinical symptoms. Statistical significance was determined at a *p*-value threshold of <0.05.

## 3 Results

### 3.1 Molecular investigation results

Out of the initial 389 oropharyngeal swabs collected, 10 were considered unsuitable for analyses due to the insufficient sample volume. For this reason, 379 samples were examined, 34 of which tested positive in the real-time PCR to screen for the presence of *Chlamydiaceae* DNA, with threshold cycle (Ct) values ranging between 25.1 to 40.0 (mean Ct = 34.7). These samples were further analyzed using the species-specific real-time PCR assay for *C. felis*.

Among the 34 samples, 27 tested positive for *C. felis* through the species-specific molecular assay. The remaining seven samples underwent additional testing with the end-point PCR targeting a 16S fragment and sequencing. Only two of the seven samples were successfully amplified and sequenced, and sequence analysis confirmed 100% identity with *C. felis* sequences present in the GenBank database (https://www.ncbi.nlm.nih.gov/ accessed on 11/07/23).

The remaining five samples resulted positive only in the initial family-specific molecular assay with Ct values above the cutoff of Ct = 35.0 (Ct ranging between 39.0 and 40.0), while they turned out to be negative in both typing assays. These five samples were classified as negative for *Chlamydia* spp. due to the possibility of cross-reactions or non-specific amplifications, as recommended by diagnostic guidelines.

### 3.2 Prevalence and risk factor analysis

Out of the 379 stray and colony cats tested, 29 were confirmed positive for the presence of *Chlamydiaceae* DNA, with all the animals infected with *C. felis.* The prevalence in the population tested totaled to 7.7% (CI95% 5.2–10.8). Epidemiological information, *C. felis* infection status and results of Pearson’s χ^2^ test are summarized in Table 1. The analysis revealed that sex, sterilization status and sampling season were statistically significant (*p* < 0.05) and associated with the infection status of the animal.

**Table 1:** Demographic and clinical information, and C. *felis* infection status of the enrolled animals.

		Positive/total (29/379)	Prevalence [95% CI] (7.7% [5.2–10.8])	*p*-value
Stage of life	Kitten	4/38	10.5% [2.9–24.8]	0.633
	Young	19/266	7.1% [4.4–10.9]	
	Adult	2/14	14.3% [1.8–42.8]	
	Senior	0/4	0% [0–60.2]	
Sex	Female	10/195	5.1% [2.5–9.2]	0.030*
	Male	19/168	11.3% [6.9–17.1]	
Sterilized status	Intact Female	6/141	4.2% [1.5–9.0]	0.025*
	Intact Male	16/134	11.9% [6.9–18.7]	
	Sterilized Female	4/53	7.5% [2.1–18.2]	1.000
	Neutered Male	3/34	8.8% [1.9–23.7]	
Season	Spring	17/113	15.0% [9.0–23.0]	0.004**
	Summer	4/85	4.7% [1.3–11.6]	
	Autumn	3/110	2.7 [0.6–7.8]	
	Winter	5/71	7.0 [2.3–15.7]	
Origin	Bozen	2/11	18.2 [2.3–51.8]	0.160
	Padua	3/64	4.7 [1.0–13.1]	
	Rovigo	7/58	12.1 [5.0–23.3]	
	Trento	2/41	4.9 [0.6–16.5]	
	Venice	9/130	6.9 [3.2–12.7]	
	Vicenza	2/53	3.8 [0.5–13.0]	
	Verona	4/22	18.2 [5.2–40.3]	
Symptomatology	Asymptomatic	27/321	11.7 [7.8–16.5]	0.294
	Respiratory symptoms	1/48	2.1 [0.1–11.1]	
	Other symptoms	1/10	10.0 [0.3–44.5]	

Prevalence and 95%CI (confidential intervals), the statistical relevance and inter-parametric evaluation performed with Pearson’s χ^2^ test (*p*-values) are indicated. Number of missing information are not shown. *p*-value of the Pearson’s χ^2^ test. *p* ≤ 0.05 was considered statistically significant, significance codes: *p* ≤ 0.01 “**”, *p* ≤ 0.05 “*”.

GAMs (Table 2) were used to analyze the risk factor and results further supported these findings. The sampling site, stage of life, and symptomatology did not exhibit statistically significant associations with *Chlamydiacea* infection (*p* > 0.05). Meanwhile, the sex of the animals, and the season of collection emerged as key predictors of the infection with *C. felis*. Male cats were found to have more than double the odds of being infected compared to females (*p* = 0.035). When both the sex and sterilization status of the animals were taken into consideration, intact males had 3 times higher odds of infection than intact females (*p* = 0.020). Sterilized animals did not show a statistically significant association with the infection status. Seasonal variation in infection was also notable. Cats sampled in summer (OR = 0.279, *p* = 0.027) and autumn (OR = 0.158, *p* = 0.004) had significantly lower odds of infection compared to animals sampled in spring. Using autumn as the baseline, the odds of infection in spring were approximately 6.50 times higher (*p* = 0.004).

**Table 2:** Results of the Generalized Additive Models (GAM) analyzing the relationship between the positivity to *C. felis* and the predictor variables.

Predictor	Missing values	Odds ratio	Standard error	*p* value
Stage of life	57			
Kitten		Ref.		
Young		0.654	0.580	0.464
Adult		1.417	0.929	0.708
Senior		0.001	447.61	0.978
Sex	16			
Female		Ref.		
Male		2.359	0.406	0.035*
Sterilized status	17			
Intact Female		Ref.		
Intact Male		3.051	0.495	0.024*
Sterilized Female		1.837	0.667	0.362
Neutered Male		2.177	0.735	0.290
Season	0			
Spring		Ref.		
Summer		0.279	0.576	0.027*
Autumn		0.158	0.642	0.004**
Winter		0.428	0.533	0.111
Origin	0			
Bozen		Ref.		
Padua		0.221	0.980	0.124
Rovigo		0.618	0.880	0.584
Trento		0.231	1.066	0.169
Venice		0.335	0.855	0.200
Vicenza		0.176	1.063	0.103
Verona		1.000	0.957	1.000
Symptomatology	0			
Asymptomatic		Ref.		
Respiratory symptoms		1.214	1.073	0.857
Other symptoms		0.238	1.031	0.163

For each predictor, we report the odds ratio, standard error, and *p*-value of the estimated effects, along with the number of missing values in the dataset. *p* ≤ 0.05 was considered statistically significant, significance codes: *p* ≤ 0.01 ‘**’, *p* ≤ 0.05 ‘*’.

## 4 Discussion

Several field studies have reported that the prevalence of chlamydial infection in pet cats ranges from 0 to 10% in healthy animals and from 5.6 to 30.9% in cats with conjunctivitis (15–17). The stray cat population exhibits higher positivity rates, ranging from 24.4 to 35.7% (17, 18, 20), with subgroups exhibiting conjunctivitis showing positive rates up to 65.8% (19). A previous Italian study used immunofluorescence assays to detect *C. felis* (29) both in cat colonies (prevalence of 68.4%) and owned cats (prevalence of 22.7%), reporting the widespread circulation of *C. felis* in the two cat populations, particularly elevated in enclosed cat colonies. However, the diagnostic value of serology for *C. felis* is limited given the high degree of cross-reactivity of antibodies against different *Chlamydia* species, and the persistence of the antibody response after infection. Moreover, the intracellular habitat of the pathogen and the predominantly localized epithelial infection may result in restricted antibody production (11). For these reasons, the direct detection of antigens or DNA provides the ultimate proof of a current infection, rather than the presence of specific antibodies. Consequently, the gold standard for diagnosing a *C. felis*-induced conjunctivitis is through the use of flocked swabs or cytology brushes collected from the conjunctiva, followed by molecular analysis of the samples (18). A previous Italian study by Rampazzo et al. (30) using a molecular assay reported 20% prevalence of *C. felis* in pet cats with conjunctivitis, and no asymptomatic positive cases detected. However, the investigation did not include stray animals.

While real-time PCR remains the gold standard for *Chlamydia* detection, infections caused by a variety of pathogens as in the case of feline upper respiratory disease (39), end-point or real-time PCR requires individual assays for each organism. In contrast, methods such as metagenomics can potentially identify all pathogens present in a sample simultaneously. Clinical metagenomics is a newer technique that employs targeted sequencing to reveal all pathogens of interest in clinical samples, while also providing their genomic information (40). A targeted metagenomics assay has recently been developed for feline upper respiratory disease (41). However, its sensitivity for detecting *C. felis* was lower compared to real-time PCR assay. This may stem from the assay design, or from the target employed. Indeed, the real-time PCR method used targeted all *Chlamydia*, whereas the target of the clinical metagenomics assay focused on a specific *C. felis* sequence. Given that cats can be infected with other *Chlamydia* species (13, 14), an approach with a *Chlamydiaceae*-specific assay to initially screen the samples, it is important to ensure a broader detection.

In our study, colony and stray cats in northeastern Italy showed a 7.7% (CI95% 5.2–10.8) prevalence of *Chlamydiaceae* infection, a percentage comparable to the one reported in previous surveys involving asymptomatic cats in Switzerland (18). All the infected animals were positive with *C. felis*, which is the most common *Chlamydia* species found in cats and a significant cause of feline acute and chronic conjunctivitis (7). The standard treatment for *C. felis* infection is doxycycline (7), and both live or inactivated vaccines are commercially available. It is important to note that the implementation of treatments, diagnostics, and prevention strategies may be challenging or ineffective in shelter or colony environments (42).

A limitation of this study is the choice to analyze oropharyngeal swabs rather than nasal/conjunctival swabs, which are considered more specific for *C. felis* infection. This could have negatively conditioned the estimated prevalence. However, a previous study did not find a significant difference in the molecular detection of *C. felis* from ocular, oropharyngeal, nasal and tongue swabs (43). Additionally, *C. felis* has already been detected using owner-collected buccal swabs for the analysis (16). For this reasons, we decided to optimize the collection process for multiple diagnoses within the same research project while minimizing the stress for the cats, considering the oropharyngeal swab as the best compromise. Another limitation of this study is that real-time PCR results, particularly those with high Ct values, only confirm the presence of *C. felis* DNA in the investigated tissue, without providing information on the pathogen viability. The step of cultivation in cell culture necessary to confirm the viability of *C. felis* is challenging and has a low sensitivity. Therefore, the presented data do not allow us to conclude whether the sampled cats were infectious and able to pose a risk to humans and other cats.

Statistical analysis revealed a higher risk of infection with *C. felis* in intact male cats and during the spring season, suggesting that behavior may influence the spread of infection. Intact male cats tend to roam more widely than females, frequently engage in fights with other tomcats to defend their territory (44), and have close contacts with female cats during the mating period, which is more common between February and June (45). This typical behavior of tomcats leads to a close contact with multiple cats, potentially increasing the risk of infection with pathogens, including *C. felis*. However, previous studies have yielded conflicting results regarding the predisposition of sex, with only one study reporting a significantly higher prevalence in male cats (46). It has been reported that the chlamydial occurrence in cats is typically higher in spring and summer, and more common in younger cats (47). In contrast, a recent study in Shanghai (22) found that *C. felis* infection in urban asymptomatic cats had a peak detection in summer and winter, with the highest positivity rates in winter, and a statistically significant difference among the seasons. Our findings corroborate that chlamydial infection have a seasonal influence; however, spring seems to be the season with the higher prevalence of infected cats. The lack of statistical significance regarding age in our study may reflect the sampling bias toward younger cats in TNR programs, which comprised 72% of the study population. The presence of symptoms, particularly conjunctivitis, have been associated with *C. felis* infection in various studies (18–20). Contrary to expectations, clinical symptoms were not associated with infection in our study. Only one of the positive cats presented respiratory symptoms, and no positive cats displayed signs of conjunctivitis. These findings highlight the need for further targeted sampling of symptomatic cats, to gain a deeper understanding of the prevalence of *C. felis* infection among the symptomatic feline population in Italy.

Although documented cases of zoonotic transmission of *C. felis* are uncommon, cats have a relatively high prevalence of *C. felis* and their proximity to humans may pose a risk. Moreover, there are reports of infection in dogs (21), and the ubiquity of cats and dogs as well as their close interactions with humans may facilitate the dissemination of *C. felis*. Indeed, sero-epidemiological studies have detected a high prevalence of antibodies against *C. felis* in humans: 1.7% of the general population and 8.8% of small animals veterinarians in Japan (20), and a prevalence of 7.6% in the Italian population (48). Infection with *C. felis* in humans typically does not result in severe disease in non-immunocompromised individuals, and it is likely that the infection is frequently overlooked or undiagnosed. Nevertheless, cat owners and professionals working with cats should pay attention, particularly when handling animals exhibiting symptoms (11).

### 5 Conclusions

In conclusion, our study demonstrates the circulation of *C. felis* in the stray cat populations of northeastern Italy, including asymptomatic individuals. This underscores the potential risk of exposure to this pathogen for humans and free-roaming domestic cats. Although the zoonotic risk is low, precautions should be taken when handling stray and colony cats, particularly among volunteer staff and professionals. Further studies are required to gain deeper understanding of the epidemiology of *C. felis*. This should include sequence analyses to implement the current knowledge on the strain circulating in Italy, as well as a strategic sampling approach based on the risk factors evidenced in our study.

## Data Availability

The original contributions presented in the study are included in the article/supplementary material, further inquiries can be directed to the corresponding author/s.
